# Mapping data elements to terminological resources for integrating biomedical data sources

**DOI:** 10.1186/1471-2105-7-S3-S6

**Published:** 2006-11-24

**Authors:** Fleur Mougin, Anita Burgun, Olivier Bodenreider

**Affiliations:** 1 EA 3888, IFR 140, Faculté de Médecine, Université de Rennes I, France; 2 National Library of Medicine, Bethesda, Maryland, USA

## Abstract

**Background:**

Data integration is a crucial task in the biomedical domain and integrating data sources is one approach to integrating data. Data elements (DEs) in particular play an important role in data integration. We combine schema- and instance-based approaches to mapping DEs to terminological resources in order to facilitate data sources integration.

**Methods:**

We extracted DEs from eleven disparate biomedical sources. We compared these DEs to concepts and/or terms in biomedical controlled vocabularies and to reference DEs. We also exploited DE values to disambiguate underspecified DEs and to identify additional mappings.

**Results:**

82.5% of the 474 DEs studied are mapped to entries of a terminological resource and 74.7% of the whole set can be associated with reference DEs. Only 6.6% of the DEs had values that could be semantically typed.

**Conclusion:**

Our study suggests that the integration of biomedical sources can be achieved automatically with limited precision and largely facilitated by mapping DEs to terminological resources.

## Background

### Introduction

The interpretation of experimental data generally requires physicians and biologists to compare their clinical and biological data to already existing data sets and to reference knowledge bases. For example, starting from a gene involved in a pathological condition, users may want to obtain information about this disease (e.g., manifestations, genes involved) and about the gene (e.g., sequence, polymorphism, pathways). This kind of information is often present in electronic biomedical resources available through the Internet. However, collecting information manually is slow and error-prone, which is essentially incompatible with high-throughput analyses. The integration of biomedical resources has been proposed as a solution to facilitate access to multiple, heterogeneous resources [1,2]. Most biomedical systems have been developed independently of each other and do not have a common structure or even a shared data dictionary. In practice, the major barriers to data sources integration are the heterogeneity of database schemas and the disparity of data elements across systems. Data elements (DEs) can be defined as a basic unit of information, also called attribute in database parlance, and which is built on standard structures having a unique meaning and distinct units or values, also called instances in databases [3]. Examples of DEs in the biomedical domain include **Gene Symbol** and **Pathology Name**. The DEs extracted from various resources tend to be heterogeneous. In fact, each source has its own way of naming the DEs it uses. For instance, a DE for pathological conditions will be named **Disorders** in one source, but **Disease** in another. In these cases, lexical approaches to integrating DEs across data sources are therefore likely to perform suboptimally. Additionally, in some sources, DEs are ambiguous because they may acquire part of their meaning from the context. For example, the DE **Name** may refer to gene or protein names. In contrast, other sources use fully specified names for their DEs, e.g., **Protein Name**. The issue here is that **Name** in protein context cannot be mapped automatically to **Protein Name** (fully specified). Conversely, two DEs **Name **in gene and protein contexts respectively must not be mapped.

The following scenario illustrates how integrating DEs facilitates the integration of biomedical sources. We want to help a biologist interested in the interactions of a given protein to query distributed sources seamlessly. To this end, the query term *interaction *has to be mapped to DEs of distinct sources: to **Interactions** in HPRD [4], to **Interactant** in Entrez Gene [5], and to **Ligand Interaction** in PDB [6]. From these resources, biologists can gain information about protein interactions in HRPD, find cross-references in Entrez Gene not only to the literature, but also to other specialized resources, such as BIND [7], and visualize chemical interactions in PDB.

The first objective of this study is to compare the DEs extracted from biomedical electronic resources to concepts and/or terms of biomedical controlled vocabularies on the one hand and to the DEs defined by the National Cancer Institute on the other. This approach should help resolve heterogeneity existing between DEs. Additionally, as some DEs are ambiguous or underspecified, we use the values associated with such DEs to identify indirect mappings to terminological resources. Our hypothesis is that we will be able to integrate DEs from heterogeneous sources by linking them to controlled terminologies, when they are not already present in reference DE repositories. The set of DEs under investigation was extracted from eleven biomedical data sources covering genes, proteins and diseases.

### Related work

The general framework of our study is that of data source integration through schema matching. Integration issues have been largely studied over the past years, with a particular emphasis on mapping schemas of heterogeneous data sources. This process takes as input two sets of elements (attributes or DEs, values, etc) constituting two schemas and determines the relations (equivalence, subsumption, etc) existing between pairs of elements across schemas. Various approaches have been developed and categorized according to distinct criteria. A brief overview of these methods is presented below. For a detailed survey of such approaches, the interested reader is referred to [8,9].

The main difference between these methods concerns the level at which they are applied. More precisely, some approaches are situated at *schema level *whereas others lie at *instance level*. *Schema-based *approaches only exploit information existing in the schema of the sources, while *instance-based *approaches exploit information situated at the instance level, i.e., the values associated with the DEs. At both schema and instance levels, two main groups of methods are used for the mapping: lexical and structural methods.

#### Schema-based approaches

*Lexical methods *have been proposed to map DE labels by exploiting their morphology. For example, a short edit distance or a high proportion of common n-grams between two strings is indicative of lexical resemblance. Others consider DE labels as terms and use external resources to identify linguistic relations (e.g. synonymy) between them. With *structural methods*, the idea is for example to consider schemas as graphs and to apply classical approaches for comparing graphs, such as determining similarity between nodes sharing common ancestors and descendants [10].

#### Instance-based approaches

The information available about schemas is sometimes insufficient or ambiguous, and it can be useful to exploit information situated at the instance level. *Lexical methods *can be used here too, for example, mapping instance values to external resources. In practice, external resources can help identify synonymy between values of attributes. *Structural techniques *can also be applied at this level where information can be obtained about constraints existing on attributes by identifying for example the range of their associated values in case of numerical data or recurring terms for textual data.

#### Examples of data source integration systems

The different kinds of methods (lexical and structural) and levels at which they are applied (*schema *and *instance*) are generally more powerful when used in combination. The system GLUE [11] provides a semi-automatic method for mapping schemas of heterogeneous sources. It combines machine learning techniques applied at the *instance level *with structural methods situated at *schema level *and which exploit the neighbourhood of attributes to determine the best mappings existing between elements of two schemas. Also of interest, [12] focuses on an *instance-based *approach and uses a domain ontology to identify indirect mappings between attributes through external knowledge, as described above. This approach is automatic but the problem is that it requires a fully specified domain ontology (with concepts well-labelled and associated instances), which represents a significant limitation.

The methods used in our study are traditional in the sense that we mostly apply lexical approaches to *schema- *and *instance-based *approaches, also taking advantage of external resources. Rather than to propose new methods, the contribution of this paper is to evaluate the applicability of existing approaches to the automatic mapping of DEs from the perspective of integrating biomedical data sources in a high-throughput context.

## Materials and methods

### Data elements

#### Origin

Our test set consists of DEs extracted from eleven Web-accessible biomedical sources, selected to be representative of the different kinds of resources found in the biomedical domain. Some of them contain information about genes: GeneCards [13], Entrez Gene, Geneloc [14], Genew (the HGNC [15] database), and HGMD [16], others about proteins: Swiss-Prot [17], PDB, HPRD, Interpro [18] or diseases: OMIM [19]. Our application is not targeted to a particular model organism so we also included MGI [20], which provides various kinds of information about mice.

#### Extracting data elements

##### Creating a set of terms for querying sources

In order to query the various data sources mentioned above, we first established a list of query terms, namely gene and disease names. To this end, we exploited a reference resource in the domain of medical genetics: the Genetics Home Reference [21] (GHR). GHR provides information about genetic conditions and genes involved in these conditions. Using the Web interface to GHR, a bioinformatician (FM) manually constituted a text file containing gene symbols (e.g. HFE) and associated disease names (e.g. hemochromatosis), if any. A sample of one hundred terms randomly extracted from this file constitutes the set of terms we used for querying DE sources.

##### Acquiring DEs

**Figure 1:**
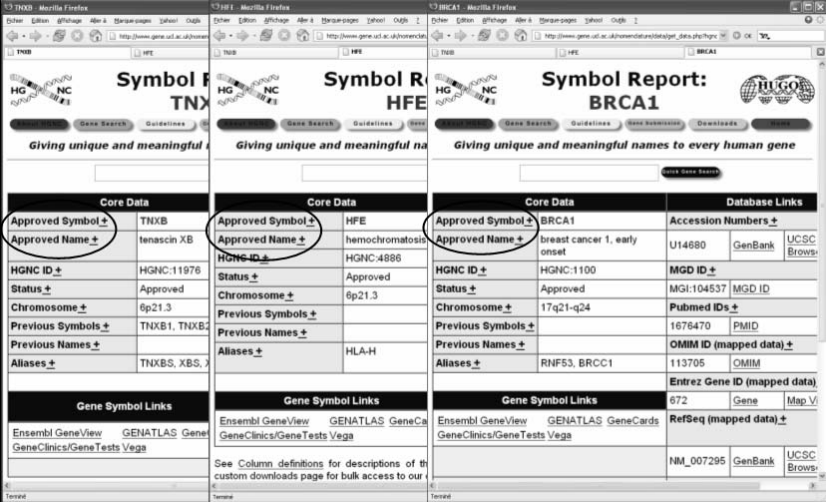
**Example of the three Genew Web pages for the TNXB, HFE, and BRCA1 genes**. Examples of data elements are encircled (Approved Symbol, Approved Name)

The sources used in this study are Web-interfaces to biological databases, automatically generated by program. Therefore, it is expected that most pages of a given source share a common organization and presentation. We take advantage of this feature for identifying recurring terms throughout Web pages, which, we hypothesize, correspond to DEs. In practice, we developed a program for querying systematically the eleven sources through their query URL. For each source, a set of 100 HTML pages corresponding to entries of the set of biomedical terms is created. After eliminating the header and footer, the elements common to at least 75% of the HTML pages are extracted automatically. This selection results in eliminating specific information (e.g., a given gene name), while keeping general information (e.g., the term "Gene Name") [22]. An example of DE extracted from the source Genew is given in Figure 1. For instance, the terms "Approved Symbol" and "**Approved Name**" appear on all three pages and are therefore identified as candidate DEs.

### Terminological resources

#### A biomedical controlled terminology: the UMLS

We chose the Unified Medical Language System^® ^(UMLS^®^) [23], a biomedical terminology integration system, because it provides a wide coverage of the biomedical domain, including terminologies for specialized clinical disciplines, the biomedical literature, and genome annotations. The UMLS consists of three major components. The UMLS Metathesaurus is assembled by integrating more than 100 sources vocabularies. It contains about 1.2 million concepts (clusters of synonymous terms) and more than 22 million relationships between these concepts. The UMLS Semantic Network is a limited network of 135 semantic types. Each Metathesaurus concept is assigned to at least one semantic type. Finally, the Lexical Resources comprise the SPECIALIST Lexicon and Lexical Tools [24]. The UMLSKS Developer's API also provides various methods for identifying Metathesaurus concepts from input terms (exact and normalized match). Additionally, the MetaMap Transfer (MMTx) program maps text to concepts in the Metathesaurus with additional flexibility (approximate match) [25]. The 2005AA version of the UMLS is used in this study.

#### A biomedical collection of data elements: the NCI caDSR

The National Cancer Institute (NCI) has created a Cancer Data Standards Registry (caDSR) [26] as part of the caCORE, a common infrastructure for cancer informatics [27]. Its main goal is to define a comprehensive set of standardized metadata descriptors for cancer research terminology used in information collection and analysis. Various NCI offices and partner organizations have developed the content of the caDSR by registration of DEs based on data standards, data collection forms, databases, clinical applications, data exchange formats, UML models, and vocabularies. Using the ISO/IEC 11179 [28] model for metadata registration, information about names, definitions, permissible values, and semantic concepts for common data elements (CDEs) have been recorded. In this study, we used the version 3.0.1.2 of the NCI caDSR, which comprises some 13,000 CDEs.

### Method

Our method can be summarized as follows. Starting from the DEs automatically extracted from eleven Web resources, we first attempt to find a direct correspondence between our DEs and biomedical terms in the UMLS on the one hand and existing CDEs in the NCI caDSR on the other. Alternatively, we map *the values *corresponding to our DEs to the UMLS and expect to determine the type of the DE using the semantic types of the terms corresponding to the DE values. More formally, we first apply lexical methods in order to map DEs extracted from distinct sources to common vocabularies by exploiting the *schema *level. We then apply lexical methods at the *instance *level and we use external resources to enhance, filter and precise DE mappings.

#### Direct mapping of data elements to terminological resources

##### Mapping to the UMLS Metathesaurus

Our approach to mapping DEs to UMLS concepts is as conservative as possible. We first attempt to find an exact match. If none is found, a match is attempted after normalization. In practice, this process makes the input and target terms potentially compatible by eliminating such inessential differences as inflection, case, underscore and hyphen variations, as well as word-order variation [24]. These two steps are implemented by the corresponding methods of the UMLSKS API. Finally, an approximate match is attempted using MMTx (strict model). The mapping procedure is fully automated and stops as soon as a match is found. The output of the mapping consists of the list of Metathesaurus concepts corresponding to each DE, along with their semantic types.

##### Mapping to the NCI caDSR

The procedure used to map DEs to the caDSR is somewhat similar to the mapping to the UMLS. The major difference is that we used a local copy of the caDSR instead of the tools provided by the NCI. This gives us additional control over the mapping process. The caDSR repository consists in twelve fields. Half of them contain numbers and other data types unlikely to map to DEs, e.g. CDE identifiers such as "2178687". Four other fields are incomplete or contain information in natural language (such as a CDE definition "The name of the gene"), they are thus difficult to exploit. In practice, out of the twelve fields in a caDSR record, only two are of interest for our purpose: "Long Name" and "Preferred Name". The corresponding values of these two fields for the CDE "Gene Name" are "GeneName" and "Name", respectively. We rendered input terms and caDSR CDEs compatible by removing spaces in multi-word terms in order to match the naming conventions in the caDSR. We first try to map exactly each DE against the Preferred Names of the caDSR. In case of failure, we attempt an exact match to the Long Names of the caDSR CDEs. Additionally, we split each multi-word DE not yet mapped to the caDSR and attempt an exact match against the Preferred Names of the CDEs, followed by an approximate match. Finally, we attempt to map exactly the isolated words from DEs to the Long Names of the caDSR CDEs. This process is also fully automated and results in a list of DEs associated with the Long Name or Preferred Name of the mapped CDE(s).

#### Indirect mapping of data elements through their values

The approaches presented in the previous section are efficient to associate DEs with lexically similar entries in the terminological resources, but they are limited to those cases where lexically similar terms exist on both sides. The alternative approach proposed here consists in mapping not the DEs, but the values associated with them to terminological resources. This indirect mapping is attempted for all DEs because the objective of the proposed approach is twofold: On the one hand, to identify mappings for those DEs for which no match in the UMLS or caDSR can be found; on the other, to filter out potential inappropriate mappings obtained through the UMLS or the caDSR. For instance, the DE **Approved Name** in Genew will be mapped to the DE **Protein Name** in SwissProt because they share the word "Name". This is incorrect because **Approved Name** actually refers to gene, not protein names. In practice, it is expected that the DEs will be found among the high-level categories characterizing their corresponding values. For example, values associated with the DE **Approved Name** include "tenascin XB", and "breast cancer 1, early onset" (see Fig. 1), categorized as *Gene or Genome*.

##### Acquiring DE values

We first created a program to automatically query each source and recovered the values associated with each DE identified in this source. We extracted automatically up to 100 values corresponding to each DE by querying the sources for each biomedical term of the set constituted as described in the paragraph ***Acquiring DEs ***of subsection Data elements. For example, the values associated with **Function** include "protein binding" and "enzyme regulator activity". In some cases, no value could be extracted for a given DE in a given source.

##### Mapping DE values to the UMLS

We used the automated methods described in the paragraph ***Mapping to the UMLS Metathesaurus ***above for mapping DE values to UMLS concepts, with the difference that only exact and normalized matches were used here. For example, **protein binding** was mapped to the concept "Protein Binding" (C0033618), categorized by the semantic type *Molecular Function*.

##### Extracting DE candidates

**Figure 2:**
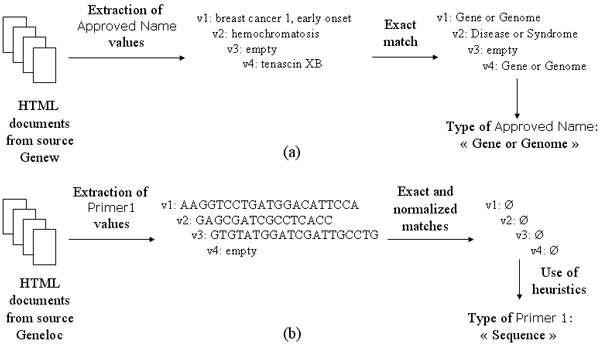
Examples of the exploitation of the values of two data elements: (a) using the UMLS as a terminological resource, (b) using heuristics.

We used the semantic type(s) of the UMLS concepts resulting from the mapping of the values of a given DE to determine the type of this DE. More precisely, we selected the semantic type categorizing the majority of the concepts for a given set of values. For instance, in the example introduced previously, we are able to determine that the DE **Approved Name** relates to gene names since the majority of its values were categorized by the semantic type *Gene or Genome *(see Fig. 2.a).

#### Default indirect mapping through data element values and heuristics

When the previous process could not determine the type of a DE, we attempted to assign coarser predefined types. We first isolated DEs containing specific terms. For instance, when the terms "ID(s)" or "identifier" were found, the corresponding DE was typed as *Identifier*. Then, we analyzed the values characterwise and assigned the type *Sequence *to the DE when each of its non-empty values was a series of "A", "G", "C", and "T". Finally, the remaining DEs were typed as *Integer *or *String *according to their values. An example of the exploitation of DE values through heuristics is shown in Figure 2.b.

This indirect mapping associates a type with the DEs, which is often useful for disambiguating underspecified DEs and for filtering out potentially inappropriate mappings obtained by direct mapping to terminological resources. Additional mappings can also be identified by exploiting the type associated with DE values, when the DE itself cannot be found in existing terminological resources.

## Results

### Disparity of DEs

474 distinct DEs (548 tokens) were extracted from the eleven selected sources, of which 47 appear in more than one source (ignoring case differences). The most frequent DEs are **Name** and **Symbol**, which appear each in six different sources.

### Direct mapping of data elements to terminological resources

For both UMLS and caDSR, we obtained different kinds of mappings. Indeed, as a DE consists of a word or a set of words, the cardinality of the mappings is either 1-1 (one DE to one UMLS concept/caDSR CDE) or 1-n (one DE to many UMLS concepts/caDSR CDEs).

#### Mapping to the UMLS Metathesaurus

391 DEs (82.5% of all distinct DEs in our set) were mapped to 479 distinct concepts of the UMLS Metathesaurus. Table 1 shows the number of DEs mapped during each step, along with the numbers of the concepts mapped to these DEs. In addition, we give two examples of DEs for the different cases. Each mapping was reviewed manually by the first author. The validity of the mappings to the UMLS is nearly 66%. Incorrect mappings occur when general terms are given a biomedical interpretation. For instance, the DE **external links** is mapped to the UMLS concept "Link" (C0208973), which is a *Pharmacologic Substance*. In fact, the DE refers to "link" in a computer-science meaning, i.e. a cross-reference. Other errors are due to the ambiguity of abbreviations, a classical issue in mapping. For example, the DE **previous GC identifiers** is mapped to the concept "GC Gene" (C1367452), while GC stands, in fact, for GeneCards. We also considered the repartition in terms of semantic types of the results obtained by our method (Table 2). This gives us an idea of what kind of information DEs represent. Not surprisingly, the semantic type under which many concepts are categorized is *Intellectual Product*, corresponding to generic concepts such as **Synonyms**, **Nomenclature**, and **database**. The semantic categorization of the DEs also helps assess the quality of the mapping (e.g., mappings of DEs to medical devices would be suspicious).

#### Mapping to the NCI caDSR

354 DEs (74.7% of all distinct DEs in our set) were mapped to 2,735 distinct DEs of the caDSR (Table 3). By exact match to the Preferred Names, we obtained 10 correct mappings, such as **gene function**. Exact match to the Long Names resulted in mapping 22 DEs to 285 caDSR CDEs. Some mappings were correct, e.g. **Location** which mapped uniquely to "MapLocation", but others were not useful in practice, such as Description which mapped to 23 distinct CDEs. After splitting multi-word DEs, ten mappings were identified by exact match to the Preferred Names, but resulted in partial matches. For instance, the DE **other accession ids** was only mapped to the caDSR CDE "other", which is incomplete and thus irrelevant. Approximate match to the Preferred Names resulted in the mapping of 273 DEs to 2,467 distinct caDSR CDEs. For example, the DE **Name** was approximately mapped to 374 distinct caDSR CDEs through the Preferred Names field. On the other hand, the approximate match to the Long Names resulted in 39 DEs mapped to 218 distinct caDSR CDEs. We did not evaluate the mappings resulting from approximate matches because of the large number of caDSR CDEs associated with each DE in these cases.

### Indirect mapping of data elements through their values and default mapping through heuristics

We analyzed the whole set of DEs. Interestingly, this method enables us to identify as distinct those lexically identical DEs whose associated value sets are different.

Overall, only 62 DEs (11.3% of all DEs in our set) could be characterized with datatypes more specific than *String*. 36 DEs were categorized by UMLS semantic types and three categories of proposed mappings were identified:

- **Correct **(11). An example is the DE **Previous symbols**, extracted from the source Genew. 90% of its 46 non-empty values were categorized by the semantic type *Gene or Genome*. We were thus able to determine that the **Previous symbols** DE in the context of the Genew source corresponds to previous gene symbols. Other examples include **Function** and **Component**, extracted from MGI, whose values are categorized by the semantic types *Molecular Function *and *Cell Component*, respectively.

- **Ambiguous **(21). For instance, the DE **Name**, extracted from the source Entrez Gene, is mapped to the semantic types *Gene or Genome *and *Amino Acid, Peptide, or Protein*, reflecting ambiguity in the UMLS. In other words, many values associated with the DE **Name** indeed correspond to both genes and proteins. For example, the value "BRCA1" maps (by exact match through synonyms) to both a protein name (BRCA1 Protein – C0259275) and a gene name (BRCA1 Gene – C0376571).

- **Erroneous **(4). Some terms were wrongly extracted from the sources. For example, **Not applicable** is extracted from the source GeneCards because it is present in many pages, but does not correspond to a DE.

The remaining DEs (26) were accurately assigned to the coarser types *Integer*, *Identifier*, and *Sequence*. Examples include:

- *Integer*: **Molecular Weight**, a DE extracted from SwissProt whose values include 207721 and 464456 (in Dalton) for BRCA1 and TNXB genes, respectively.

- *Identifier*: **Accession Numbers**, a DE extracted from Genew whose values include U14680 and X71923 (GenBank identifiers) for BRCA1 and TNXB genes, respectively.

- *Sequence*: an illustration of **Primer 1** extracted from GeneLoc is given in Figure 2.b.

Table 4 shows the number of DEs associated with the various datatypes.

### Examples

We present here two examples illustrating the whole mapping process: results obtained by direct and indirect approaches are displayed. The two DEs we have chosen are **From **extracted from the source SwissProt and **RT-PCR** obtained from MGI.

#### Direct mapping

##### UMLS Metathesaurus

The DE **From** is not found in the UMLS whereas the abbreviation RT-PCR maps unambiguously (by exact match through a synonym) to the concept "Reverse Transcriptase Polymerase Chain Reaction" (C0599161).

##### NCI caDSR

Both DEs are found through an approximate match to the field Preferred Name. The DE **From** results in eight CDEs, such as "ExternalReferenceExportedFromS". **RT-PCR **maps to three CDEs including RT-PCR_RESULT_PROC, which corresponds to the results obtained by the RT-PCR procedure. None of the mappings obtained through approximate match are accurate.

#### Indirect mapping

##### Semantic types

94 non empty values are retrieved from the DE **From**, including "Rattus norvegicus", "Zebrafish", and "Homo sapiens". Among those, 47.9% are categorized by *Mammal *and 11.7% by *Fish *(and 100% by the more general semantic type *Organism*), thus indicating that this DE refers to organisms. Therefore, we can identify an indirect mapping to the DEs **Organism** present in Entrez Gene and GeneCards.

##### Heuristics

The indirect mapping of **RT-PCR **requires the use of heuristics. The values of this DE are digits, which corresponds to the number of RT-PCRs realized on the gene whose information Web page is displayed on MGI Web site. The associated type is thus *Integer*.

## Discussion

### Findings

#### Direct mapping

Intuitively, mapping to a reference DE repository represents the best possible data integration approach. This intuition was confirmed in part by this study as illustrated by the following example. The DE **Gene Name** exists in the caDSR, where it is related to the more generic CDE "Gene". In our experience, however, beside a limited number of such mappings (only 10 are deemed correct), this approach was rather ineffective because most of our DEs could not be found in the caDSR. Moreover, the approximate matching often yielded too many candidates to be useful in an automated environment. In contrast, the mapping of DEs to the UMLS turned out to yield the majority of the mappings. The broad coverage provided by the UMLS Metathesaurus explains the large number of exact matches. Approximate matches, while useful for guiding the mapping, are of limited interest in an automated environment. For example, there is no exact or normalized match in the UMLS for the DE **Gene Name** and this DE is mapped to the two concepts "Gene" and "Name". The mapping to "Name" is too generic and would result in ambiguity with other DEs such as **Protein Name**. Analogously, **Gene Name** and **Gene Symbol** cannot be easily differentiated if the mapping to "Gene" is selected.

#### Indirect mapping

Because our method selects the semantic type common to most values for a given DE, it achieves a semantic typing of the DEs rather than a real mapping. In fact, the direct and indirect mappings of DEs are complementary. Direct mapping identifies a direct correspondence between DEs through existing terminological resources, whereas indirect mapping is useful for disambiguating mappings. As illustrated before, we were able to indicate that the DE **Approved Name** is to be understood in the context of genes (i.e., gene name) and that the DE **From** represents the organisms in which a protein is expressed. However, overall, only 6.6% of our DEs could be semantically typed by this method.

### Partially automated mapping

The purpose of semantic mining is to identify and characterize the relations among entities of interest in a given domain. Because biomedical knowledge is scattered across many heterogeneous databases, data integration is often used in semantic mining applications. Moreover, semantic mining techniques are usually applied in high-throughput environments, where manual data integration is impractical. Our results suggest that data integration can be achieved automatically with limited precision and largely facilitated by mapping DEs to terminological resources. Our approach exploits both *schema *and *instance *levels for aligning schema sources, which is not new in itself. However, this study illustrates concretely the benefit of automating the mapping process for biomedical sources integration, in contrast to integration systems that are designed and maintained mostly manually. The methods presented in this paper would support the partial automation of some tasks related to the conception and evolution of integration systems. Indeed, our approach contains the ingredients of a mediator-based system [29]: information about sources (we extract DEs for that), a global schema (which can be represented using terminological resources, such as the UMLS), and finally mappings between elements of the global schema and source schemas (we present here methods for mapping DEs to entries of terminological resources).

### Limitations and future directions

#### Evaluation

In this exploratory study, the validity of the mappings was evaluated by one person only (FM). An independent evaluation would be required to confirm our results.

#### General lexical resources

Among the DEs that failed to be mapped to the UMLS and caDSR are general terms such as **Pathways**, **Ontologies**, **keywords**, **domain**, and **features**. Mapping to general rather than specialized resources is expected to compensate for this limitation. We plan to add WordNet [30], the electronic lexical database of general English, to our list of target terminological resources. We would like to evaluate the potential benefit of using general lexical resources to increase the coverage of non-domain-specific DEs, even if we are aware that using such resources will likely result in increased ambiguity for some DEs.

#### Patterns and rules

The heuristics currently used for analyzing the DE values only identify a limited number of datatypes. Pattern detection could be used to enrich some datatypes with semantic information. For example, a pattern for identifying bibliographic references would allow us to relate the DEs **Primary Citation** in PDB and **Publications** in InterPro. Analogously, rules could be used to combine multiple direct mappings. For example, a composite concept "Gene name" could be created from the mapping of the DE **Gene name** to the two UMLS concepts "Gene" and "Name".

## Conclusion

The aim of our study was to consider the integration of biomedical sources through the use of DEs. We extracted a set of DEs from disparate biomedical sources available on the Internet. We then demonstrated the benefit of using terminological resources to reconcile heterogeneous DEs. Terminological resources were useful from a lexical perspective, enabling to map DEs to a common vocabulary. In addition, from a semantic perspective, terminological resources supported the categorization of DE values, enabling us to disambiguate underspecified DEs.

## Figures and Tables

**Table 1:** Mapping steps of data elements in the UMLS Metathesaurus

**Step**	**Number of mapped DEs/associated UMLS concepts**	**Data element**	**UMLS concept(s)**
**Exact match**	139/204	**Molecular Weight Northern Blot**	Molecular Weight (C0026385) Northern Blot (C1148548)
**Normalized match**	20/23	**cellular component molecular function**	cellular_component (C1166607) molecular_function (C1148560)
**Approximate match (MMTx)**	232/333	**Gene Symbol** **MRNA Sequence**	Genes (C0017337) Symbol (C0679214) RNA, Messenger (C0035696)


**Table 2:** Repartition of the data elements under UMLS semantic types

**Number of mapped concepts**	**Semantic type**	**Example of data element**	**Example of proposed concepts**
37	*Intellectual Product*	**Gene Name**	Names (C0027365)
34	*Body Part, Organ, or Organ Component*	**biological process**	Biological process (C1184743)
26	*Functional Concept*	**Genomic context**	Context (C0542559)
25	*Qualitative Concept*	**Mutation type**	Type (C0332307)
19	*Spatial Concept*	**site of expression**	Site (C0205145)
17	*Neoplastic Process*	**malignant neoplasms**	malignant neoplasms (C0006826)
17	*Quantitative Concept*	**sensitivity**	Statistical sensitivity (C0036667)
16	*Pharmacologic Substance*	**Drug similarity**	Drugs (C0013227)
14	*Body System*	**immune system**	immune system (C0020962)
14	*Disease or Syndrome*	**disorders & mutations**	Disease (C0012634)

**Table 3:** Results of the direct mapping of data elements to the NCI caDSR

**Step**	**DEs**	**CDEs**	**Examples of DEs mapped to CDE(s)**
**Exact match of DEs to PN**	10	10	Correct : **gene function** to "GeneFunction"
**Exact match of DEs to LN**	22	285	Correct : **Location** to "MapLocation" Not useful : **Description** to 23 CDEs (e.g. "ContextDescription" and "GeneAliasDescription")
**Exact match of split DEs to PN**	10	10	Partial : **other accession ids** to "other"
**Approximate match of split DEs to PN**	273	2,467	Not useful : **Name** to 218 CDEs (e.g. "ApprovedStatusName" and "OTHER_DZ_SITE_NAME")
**Approximate match of split DEs to LN**	39	218	Not useful : **Component** to 26 CDEs (e.g. "Protein Component" and "Blood Component Transfusion Date")

PN: Preferred Name, LN: Long Name, CDEs: common data elements

**Table 4:** Results of the indirect mapping through data element values and heuristics

**Type**	**Number of DEs having this type**	**Examples of typed DEs**
**Semantic type**	36 (6.6%)	**Previous symbols** (*Gene or Genome*)
**Integer**	18 (3.3%)	**product size**
**Identifier**	6 (1.1%)	**PubMed IDs**
**Sequence**	2 (0.3%)	**Primer 2**
**String**	486 (88.7%)	**Bibliography**
